# Extraction of Keratin from Rabbit Hair by a Deep Eutectic Solvent and Its Characterization

**DOI:** 10.3390/polym10090993

**Published:** 2018-09-06

**Authors:** Dongyue Wang, Xu-Hong Yang, Ren-Cheng Tang, Fan Yao

**Affiliations:** National Engineering Laboratory for Modern Silk, College of Textile and Clothing Engineering, Soochow University, 199 Renai Road, Suzhou 215123, China; 18896500672@163.com (D.W.); lucky940829@163.com (F.Y.)

**Keywords:** keratin, rabbit hair, deep eutectic solvent, dissolution, extraction, biopolymers

## Abstract

Keratin from a variety of sources is one of the most abundant biopolymers. In livestock and textile industries, a large amount of rabbit hair waste is produced every year, and therefore it is of great significance to extract keratin from waste rabbit hair in terms of the treatment and utilization of wastes. In this study, a novel, eco-friendly and benign choline chloride/oxalic acid deep eutectic solvent at a molar ratio of 1:2 was applied to dissolve waste rabbit hair, and after dissolution keratin was separated by dialysis, filtration, and freeze-drying. The dissolution temperature effect was discussed, and the resulting keratin powder was characterized by X-ray diffraction, scanning electron microscope, Fourier transform infrared spectroscopy, protein electrophoresis, thermogravimetry and differential scanning calorimetry, and amino acid analysis. During the dissolution process, the α-helix structure of rabbit hair was deconstructed, and the disulfide bond linkages were broken. The solubility of rabbit hair was significantly enhanced by increasing dissolution temperature, and reached 88% at 120 °C. The keratin produced by dissolving at 120 °C displayed flaky powders after freeze-drying, and had a molecular weight ranging from 3.8 to 5.8 kDa with a high proportion of serine, glutamic acid, cysteine, leucine, and arginine. Such features of molecular weight and amino acid distribution provide more choices for the diverse applications of keratin materials.

## 1. Introduction

Keratin is one of the most abundant biopolymers, and is widely distributed in animal hairs and feathers [1]. In agricultural and industrial production, a large amount of hair and feather wastes are produced every year, resulting in the loss of keratin resources and the aggravation of environmental pollution. Therefore, the recovery and utilization of keratin from animal hairs and feathers are of great significance and have become a research hotspot. As a high value-added and environmentally compatible substance, the extracted keratin is widely studied in biological materials [2], and has found its applications in many fields. The wool-based keratin hydrolyzates obtained by superheated water and alkaline hydrolysis, and with low molecular weight, were used as an amendment fertilizer to improve plant growth and soil condition [3,4]. The polypeptides of chicken feather keratin hydrolyzed by calcium hydroxide at a high temperature were used as an animal feed supplement [5]. The application of keratin peptide and protein to human hair had a restoring ability in chemically damaged hair [6]. The blends of keratin with other polymers were frequently electrospun to nanofiber mats for diverse applications [7]. Keratin was also used as an additive in natural and synthetic polymers to fabricate composite fibers and materials for improved properties [8,9]. In textile industry, the keratin hydrolyzate modified with a cationic agent was an alternative to sodium sulfate for enhancing the uptake of reactive dyes by cotton fabrics [10]; the superheated water hydrolyzed keratin was employed as a foaming agent in the foam dyeing of cotton and wool fabrics [11]; the keratin extracted by the reduction method could enhance the hydrophilic property of aminolyzed polyester fabric [12]. The different applications of keratin mentioned above have individual requirements. Usually, as an additive in composite materials, keratin with high molecular weight should be used due to the strength requirement, whereas as regarding dyeing and finishing agents, a low molecular weight keratin would be suitable because the water solubility of keratin and the softness of textiles must be taken into account. The different dissolution and extraction approaches of animal hairs and feathers produce different molecular weight keratins which can provide more choices for the diverse applications of keratin materials.

The keratin fiber has a stable three-dimensional conformation maintained by a range of noncovalent (hydrogen bonds, ionic bonds, and hydrophobic interactions) and covalent bonds (disulfide bonds) [1,13]. Keratin is distinct from other fibrous proteins in its high sulfur content, high stability of physical and chemical structures, and strong resistance to chemical attacks mainly due to inter- and intrachain cysteine disulfide bond cross-links [1,13]. As far as the crystalline structure is concerned, keratin has three structural forms: α, β, and γ keratin. α-Keratin is tightly coiled, leading to the formation of a rod-like structure, whereas β-keratin consists of a high proportion of parallel sheets of molecular chains [1]. The high stability of the keratin fiber presents a challenge for the dissolution and extraction of keratin substances. Therefore, the breakage of peptide bonds by strong acids and alkalis, the disruption of disulfide bonds by oxidizing and reducing agents, and the deconstruction of physical structures by hydrogen bond disrupters as well as their combinations must be used to dissolve the keratin fiber [14]. Of these approaches, some are limited because of the use of long time, high temperature, and harsh reaction conditions, whereas others have the shortcomings of the use of toxic and harmful reagents. In order to solve the existing problems in the current methods, novel solvents deserve to be further attempted. In this regard, the extraction of keratin by superheated water and ionic liquids has been successfully carried out, and considered as a more environmentally friendly method [13,15,16,17].

On the other hand, deep eutectic solvent (DES) as a novel and eco-friendly solvent has received great attention. DES is formed by the complexation of hydrogen bond acceptors (HBA) and hydrogen bond donors (HBD) [18]. HBA and HBD interact with each other to produce a lower freezing point, allowing them to form a stable solvent at a low temperature [19]. For instance, whereas the melting points of choline chloride and urea are 303 and 133 °C, respectively, the freezing point of their mixture at a urea to choline chloride molar ratio of 2:1 is 12 °C [18]. DES integrates the advantages of good biocompatibility, environmental protection, economy, innocuity, and harmlessness [20], and its research and application have permeated in many chemical fields, such as extraction and separation, synthesis media, metal processing, catalytic reactions, polymer dissolution, etc. [20,21,22,23,24]. Recently, the application of DES in the extraction and separation of natural proteins has also attracted interest from researchers. The DES consisting of choline chloride and urea was employed to deconstruct wool and extract keratin at 170 °C [25], and a combination of choline chloride and oxalic acid was successfully used to extract collagen peptides from cod skins at 65 °C [26].

China is an important producer of rabbit hair and Chinese rabbit hair production makes up a large proportion of world output [27]. In recent years, the application of rabbit hair in textile industry has attracted great attention. However, because of the low friction coefficient and cohesion force between rabbit hairs, rabbit hair and its mixture with other fibers have low spinnability, and the resulting yarns suffer from a shortcoming of low yielding rate [27,28], thereby producing waste rabbit hair in the spinning process. Additionally, in farms and slaughterhouses, an amount of waste rabbit hair is produced. Like other animal hairs, rabbit hair is rich in keratin which is a valuable natural protein. Thus, how to recycle the waste rabbit hair has also become a research subject.

In the previous research, a L-cystein and urea mixture was used to extract keratin from rabbit hair at pH 10.5 and 75 °C for 5 h; the resulting keratin had a molecular weight of about 11 kDa [29]. Recently, we preliminarily explored the combinations of choline chloride with urea, ethylene glycol, citric acid, and oxalic acid at 1:1, 1:2, and 1:3 molar ratios of choline chloride to other additives as DESs to dissolve waste rabbit hair at 98 °C for 2 h, and found that the solubility of rabbit hair was 19–22% in choline chloride-urea, 15–20% in choline chloride-ethylene glycol, 20–27% in choline chloride-citric acid, and 36–71% in choline chloride-oxalic acid, and the choline chloride-oxalic acid mixture at a molar ratio of 1:2 or 1:3 showed a better dissolution effect on rabbit hair. Additionally, prolonging time could increase the solubility of rabbit hair at 98 °C. In this work, the combination of choline chloride and oxalic acid at a molar ratio of 1:2 was used as DES to dissolve rabbit hair, and the effect of temperature on the dissolution efficiency of rabbit hair was discussed. Afterwards, the keratins obtained by dissolving at different temperatures were separated by dialysis, filtration, and freeze-drying. The resulting keratin powders were characterized by Fourier transform infrared (FT-IR) spectroscopy, X-ray diffraction (XRD), thermogravimetry (TG)/differential scanning calorimetry (DSC), protein electrophoresis, scanning electron microscope (SEM), and amino acid analysis.

## 2. Materials and Methods

### 2.1. Materials

Waste rabbit hair used in this paper was collected from a slaughterhouse (Shiyan, China). It was scoured in a 2 g/L sodium carbonate solution at 50 °C for 30 min, rinsed thoroughly in tap water, and then dried in open air. The resulting rabbit hair was used in the dissolution test.

Choline chloride and oxalic acid were of analytical reagent grade, and purchased from 9-Ding Chemistry Co. Ltd. (Shanghai, China) and Titanchem Co. Ltd. (Shanghai, China), respectively. The dialysis bag with a theoretical molecular weight cut-off of 3 kDa was provided by Biosharp Co. Ltd. (Hefei, China).

### 2.2. Extraction of Keratin from Rabbit Hair with DES

Choline chloride and oxalic acid were mixed at a molar ratio of 1:2 in the conical flask, and then the flask was placed in the XW–ZDR low-noise oscillated dyeing machine (Jingjiang Xinwang Dyeing and Finishing Machinery Factory, Jingjiang, China) and oscillated at 70 °C for 20 min. The resulting homogeneous and colorless solution was called DES.

Rabbit hair (0.2 g) was thoroughly immersed in DES (20 g) present in the conical flask which was placed and heated in a glycerol bath. The mixture of rabbit hair and DES was heated for 2 h under a stirring condition at various temperatures ranging from 80 to 120 °C. After cooling, the solution was dialyzed in a water bath for 2 days using the dialysis bag with a theoretical molecular weight cut-off of 3 kDa. After dialysis, the mixture was filtered to remove undissolved rabbit hair. Afterwards, the resulting undissolved rabbit hair was dried, and the solubility of rabbit hair was calculated using the following equation:

(1) $$\mathrm{Solubility}\;(\%)=\frac{W_{0}-W_{1}}{W_{0}}\times 100$$

where *W*_0_ and *W*_1_ represent the weight of rabbit hair before and after dissolution, respectively.

In order to obtain the keratin powders for further characterization, the above filtrate solution was freeze-dried using the VirTis freeze mobile 25EL lyophilizer freeze dryer (SP Scientific Inc., New York, NY, USA).

### 2.3. Measurements

The UV-Vis absorption spectra of rabbit hair keratin solutions were recorded by the Shimadzu UV-1800 UV-Vis spectrophotometer (Shimadzu Co., Kyoto, Japan). The FT-IR spectra of rabbit hair and keratin powder were recorded by the Nicolet 5700 FTIR spectrometer (Thermo Fisher Scientific Inc., Waltham, MA, USA) using KBr pellets. The amino acid analysis of rabbit hair and keratin powder was carried out using the L-8900 automatic amino acid analyzer (Hitachi High-Technologies Co., Tokyo, Japan); the sample (10 mg) was hydrolyzed with 6 mol/L HCl at 110 °C for 24 h in nitrogen atmosphere, and then the diluent (0.2 mg/mL) was used for the free amino acid analysis; the amino acid composition was determined by calibration with the external standard amino acid solutions, and each sample was analyzed in duplicate.

The molecular weight distribution of keratin was measured on the PowerPac universal type protein electrophoresis (Bio-Rad Laboratories Inc., Hercules, CA, USA) by sodium dodecyl sulphate-polyacrylamide gel electrophoresis (SDS-PAGE). According to the method depicted by Laemmli [30], 5% concentration gel and 12% separation gel were chosen as electrophoresis plastics, and the low-molecular-weight standard protein (3.8–20.1 kDa) was used as a marker for electrophoresis.

The surface morphologies of rabbit hair and its residues in DES solution as well as keratin powders were observed by the TM3030 tabletop SEM (Hitachi High Technologies America, Inc., Schaumburg, IL, USA) with an acceleration voltage of 15 kV. The XRD patterns of rabbit hair and keratin were obtained by X’Pert-Pro MPD X-ray diffraction (PANalytical B.V., Almelo, The Netherlands). The TG and DSC thermal analyses of rabbit hair and keratin powder were obtained by the SDT Q600 thermal analyzer (TA Instruments Inc., New Castle, DE, USA) in nitrogen with the temperature range from 40 to 600 °C at the heating rate 10 °C/min.

## 3. Results and Discussion

### 3.1. Dissolution of Rabbit Hair in DES

**Figure 1:**
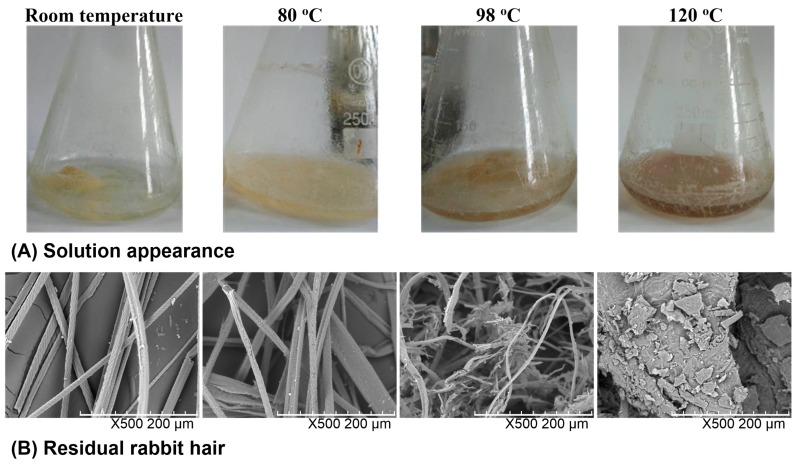
Appearance of the rabbit hair and DES mixture (**A**) and SEM images of residual rabbit hairs at various temperatures (**B**).

The solubility of rabbit hair in DES at various temperatures was observed from the quantity of residual rabbit hair in the solvent, the transparency of the solvent, and the morphological structure of residual rabbit hair. Figure 1 shows the appearance of the rabbit hair and DES mixture as well as the SEM images of residual rabbit hairs. At room temperature, rabbit hair was insoluble in DES, and the shape of rabbit hair was clearly visible with the clean scales on its surface. At 80 °C, a large number of rabbit hairs were dispersed in DES, and most of the scales on the rabbit hair surface were still present but some hairs and scales were damaged, indicating the low solubility of rabbit hair at this temperature. At 98 °C, most of rabbit hairs were broken into pieces by DES, and lost their clean fibrous shapes. At 120 °C, almost no rabbit hair fiber was found in solution, and the solution turned out to be viscous and dark brown. The increased viscosity is due to the high solubility of keratin, while the color change originates from the release of melanin in cortical cells, which is also found in the dissolution of wool by the DES of choline chloride and urea [25]. Moreover, only a small amount of residues with a sheet structure were collected. The above observations indicate that with the increase of dissolving temperature, the solubility of rabbit hair increases and at 120 °C rabbit hair approaches to complete dissolution.

### 3.2. Solubility of Rabbit Hair and Absorbance of Regenerated Keratin Solution

**Figure 2:**
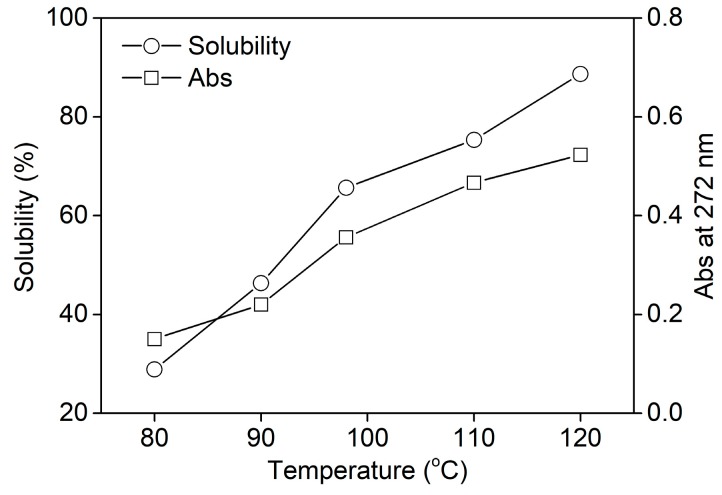
Solubility of rabbit hair in DES at various temperatures.

In order to investigate the dissolving property of rabbit hair at various temperatures quantitatively, the solubility of rabbit hair and the absorbance of keratin solution were measured and shown in Figure 2. The keratin solution had a maximum absorption peak at about 272 nm, corresponding to the absorption of the aromatic amino acid sequences [31]. Thus, the absorbance of keratin solution at 272 nm could characterize the quantity of keratin. Figure 2 shows that both the solubility of rabbit hair and the absorbance of keratin solution almost increased linearly with the rise in temperature. This reveals the enhanced soluble behavior of rabbit hair with rising temperature. At 80, 98, and 120 °C, the solubility of rabbit hair was 28.9%, 65.7%, and 88.7%, respectively. Rabbit hair exhibited high solubility at 120 °C. The increased solubility of rabbit hair at high temperatures can be explained by the following. (a) As the temperature rises, the viscosity of DES reduces, promoting the mass transfer effect between DES and rabbit hair [26]. Additionally, the swelling extent of rabbit hair increases at high temperatures. The two factors can accelerate the diffusion of DES into the hair interior and subsequently enhance the dissolution of rabbit hair. (b) With the increase in temperature, the ionization of oxalic acid increases, causing an increase in the acidity of the dissolution system. Thus the increased ionization of basic amino acid sequences weakens the interactions between protein molecules [26], and at the same time the strong acidity increases the hydrolysis extent of protein, resulting in the increased solubility of rabbit hair.

From the results of Figure 1 and Figure 2, rabbit hair has a high solubility at 120 °C in the choline chloride/oxalic acid solvent at a molar ratio of 1:2. The use of the same solvent at a choline chloride to oxalic acid molar ratio of 1:1 provides a high extraction efficiency of collagen peptides from cod skins at 65 °C [26]. The choline chloride and urea solvent at a molar ratio of 2:1 can extract keratin from wool fiber at 170 °C [25]. These facts indicate that the dissolution of keratin fibers with the cuticle layers in DESs must be carried out at high temperatures because of their tight structures.

### 3.3. Characterization of Regenerated Keratin

#### 3.3.1. Morphological Structure

**Figure 3:**
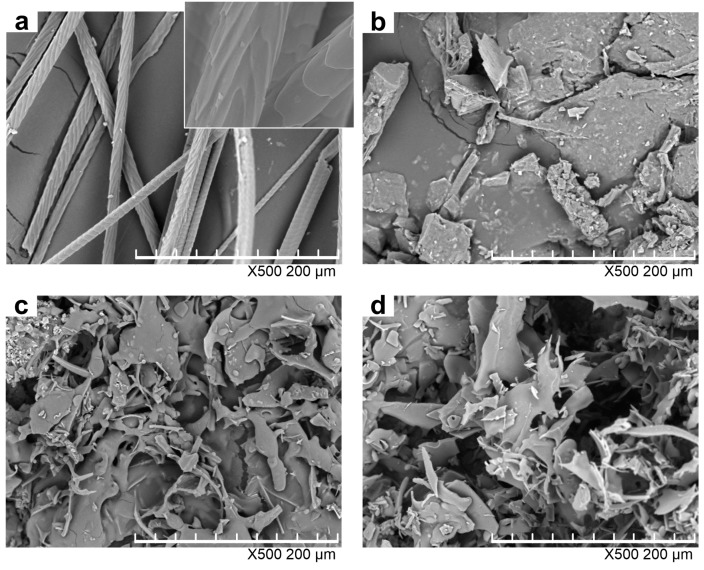
SEM images of rabbit hair (**a**) and keratin samples produced by dissolving at 80 (**b**); 98 (**c**); and 120 °C (**d**).

Figure 3 shows the morphologies of rabbit hair and regenerated keratin powders by dissolving in DES at various temperatures. With regard to the raw rabbit hair, the scales were clearly visible, and the cuticle scale edges were at large angles to the axial direction as shown in the enlarged image in Figure 3a. Compared with the raw rabbit hair, the keratin powders underwent dramatic changes in shape, and did not display the residue of rabbit hair, indicating that they lost the compact structure of rabbit hair. The keratin obtained by dissolving at 80 °C was in the form of lumpy powders, whereas the samples produced at 98 and 120 °C became flaky powders and had a loose structure as high temperatures greatly enhanced the rupture extent of rabbit hair. Such a loose structure makes the regenerated keratin easier to be applied in various fields [32].

#### 3.3.2. Molecular Weight Distribution

**Figure 4:**
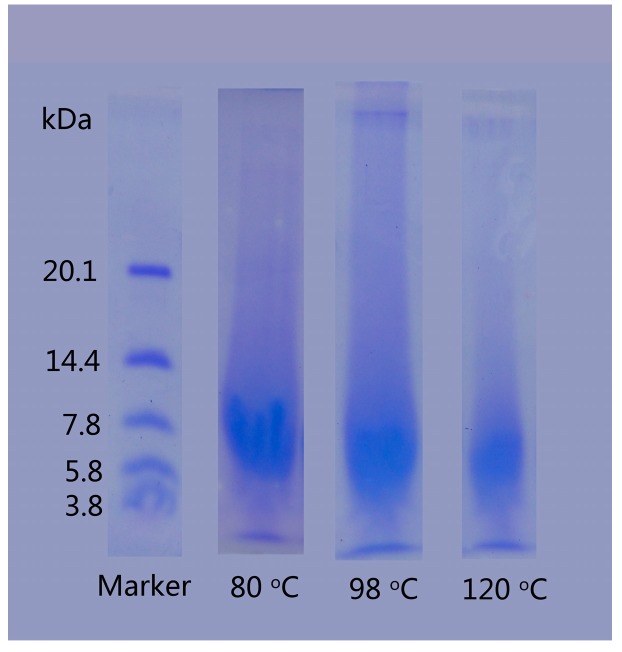
SDS-PAGE of regenerated keratins by dissolving in DES at various temperatures.

The SDS-PAGE analysis was employed to investigate the molecular weight distribution of regenerated keratins produced by dissolving in DES at various temperatures (Figure 4). The dissolving temperature had an obvious influence on the molecular weight of the keratin. For keratin produced at 80 °C, the entire band was stained, indicating that the molecular weight is distributed throughout the band and some fragments had molecular weight exceeding 20.1 kDa, but the majority had a molecular weight ranging from 5.8 to 10 kDa. The molecular weight of regenerated keratins produced by dissolving at 98 and 120 °C was between 3.8 and 7.8 kDa and 3.8 and 5.8 kDa, respectively. As the temperature increases, the decomposition degree of rabbit hair is aggravated and more keratin molecules are disintegrated to smaller pieces, resulting in the increased content of low molecular weight keratin. The lower molecular weight of regenerated keratins produced at higher temperatures is in agreement with the morphological structure of Figure 3, where the high temperature dissolution produces a more loose form of keratin.

#### 3.3.3. XRD Pattern

**Figure 5:**
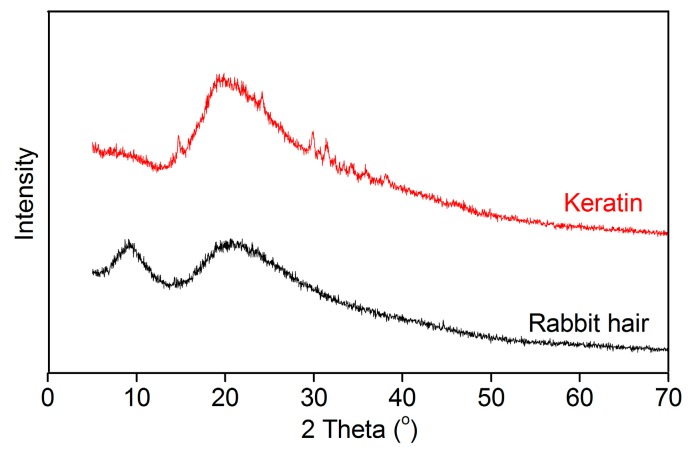
XRD patterns of rabbit hair and regenerated keratin by dissolving in DES at 120 °C.

The XRD patterns of rabbit hair and keratin obtained using DES at 120 °C are shown in Figure 5. The raw rabbit hair showed a small diffraction peak at 9° and a broad diffraction peak at 20°, corresponding to the α-helix and β-sheet structures, respectively [16]. In the XRD pattern of the extracted keratin, the diffraction peak at 9° disappeared, indicating that the α-helix structure of rabbit hair is destroyed in the dissolution process [16,29,33]. Interestingly, compared with the raw rabbit hair, the extracted keratin displayed an increase in the diffraction intensity of β-sheet structure at 20°, suggesting the increased content of β-sheet structure in keratin. These changes reveal the disorder and rearrangement of keratin crystallites during the dissolution and regeneration process. Similar changes were also found in the keratin extracted from rabbit hair using the mixture of L-cystein and urea [29].

#### 3.3.4. Thermal Stability

**Figure 6:**
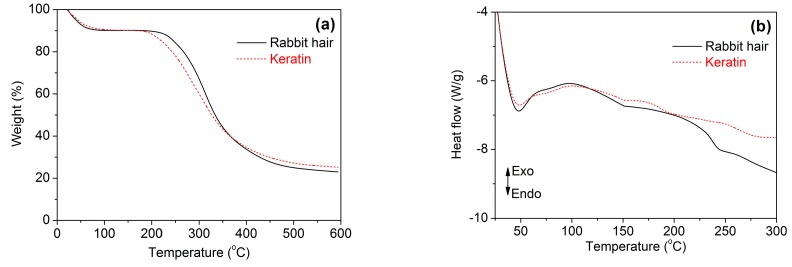
TG (**a**) and DSC (**b**) curves of rabbit hair and regenerated keratin by dissolving in DES at 120 °C.

Figure 6 shows the thermal behaviors of rabbit hair and regenerated keratin obtained using DES at 120 °C. In the TG curves of Figure 6a, both rabbit hair and regenerated keratin underwent a three-step weight loss. The first weight loss step occurred below 150 °C, corresponding to the evaporation of bound water. The second weight loss step was in the temperature range of 220 to 375 °C for the raw rabbit hair and 190 to 370 °C for the regenerated keratin, respectively, which is a rapid weight loss step caused by the melting and destruction of α-helix structure, the breakage of disulfide bonds, the thermal decomposition of peptide bridges, chain linkages, and small lateral chains, and the evolution of gaseous products [34,35]. The last step was a gradual weigh loss caused by the further pyrolysis of the degraded products. Compared with the raw rabbit hair, the regenerated keratin showed reduced thermal stability, as indicated by its lower initial degradation temperature and higher weight loss at the second weigh loss step. This poor thermal stability is mainly due to the lower molecular weight and disordered structure of regenerated keratin [36], and it is coincident with the loss of crystallinity indicated by XRD.

In the DSC curves of Figure 6b, the raw rabbit hair had an endothermic peak at 242 °C, which is assigned to the melting and destruction of α-helical crystallites [13,37]. However, the same peak disappeared for the regenerated keratin, but a weak peak with an endothermic effect appeared at approximately 196 °C. Such changes may be associated with the formation of disordered structures during the dissolution of rabbit hair at high temperature [13,37].

#### 3.3.5. FT-IR Spectrum

**Figure 7:**
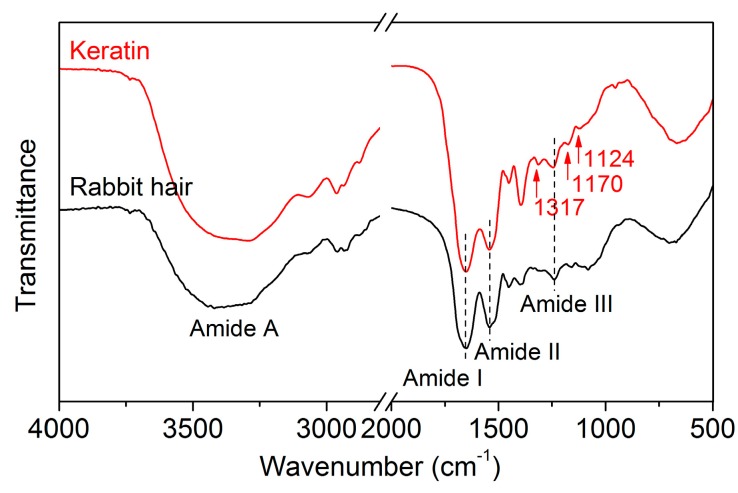
FT-IR spectra of rabbit hair and regenerated keratin by dissolving in DES at 120 °C.

The FT-IR spectra of rabbit hair and regenerated keratin obtained using DES at 120 °C are shown in Figure 7. The FT-IR spectrum of the raw rabbit hair displayed the characteristic absorption bands of keratinous fibers at 3420, 1650, 1543, 1240, and 685 cm^−1^, which are assigned to the absorption of hydrogen bonded NH groups (amide A, N–H stretching), amide I (C=O stretching), amide II (N–H bending), amide III (C–N stretching), and amide IV, respectively [1]. The regenerated keratin showed no noticeable changes in the positions of absorption bands, indicating that the DES dissolution followed by dialysis can provide a purified keratin product. Compared with the rabbit hair, the regenerated keratin exhibited the intensified absorption of the amide I band, which might be associated with a lower quantity of α-helical crystallites [1]. Additionally, three novel weak absorption bands appeared at 1317, 1170, and 1124 cm^−1^, which are related to the breakage of disulfide bonds and the formation of sulfur-containing derivatives [1,36].

#### 3.3.6. Amino Acid Composition

**Figure 8:**
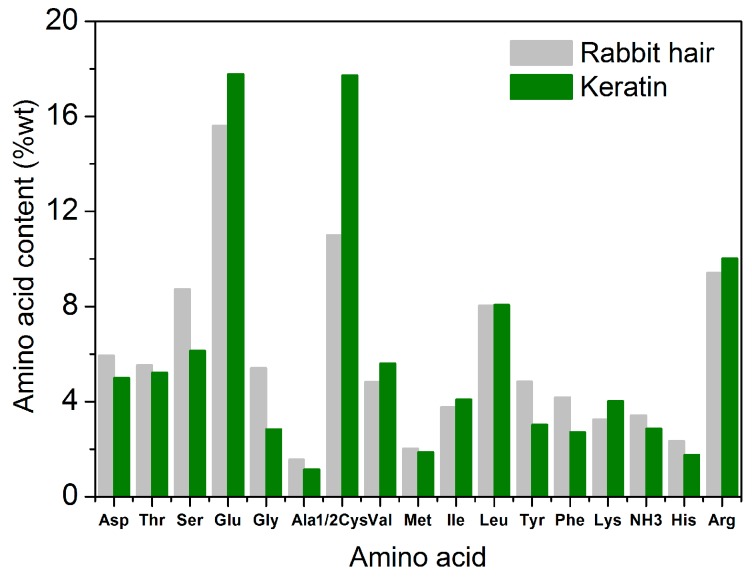
Amino acid content of rabbit hair and regenerated keratin by dissolving in DES at 120 °C.

Figure 8 shows the amino acid compositions of rabbit hair and regenerated keratin produced by dissolving at 120 °C. Regardless of rabbit hair or regenerated keratin, the main amino acids compositions were almost the same, and they were serine, glutamic acid, cysteine, leucine, and arginine. In the total amino acids analyzed in the present study, the content of the five amino acids was 52.8% for rabbit hair, and 59.8% for regenerated keratin, respectively. Compared with the raw rabbit hair, the regenerated keratin had obvious changes in amino acid compositions. The contents of aspartic acid, serine, glycine, alanine, tyrosine, phenylalanine, and histidine decreased considerably (reduction rate exceeded 15%), whereas the contents of glutamic acid, cysteine, valine, and lysine increased considerably (increment rate exceeded 13%). Glycine and cysteine had the highest reduction and highest increment in content, respectively. These changes of amino acid compositions indicate that the different acid amino acid sequences of rabbit hair are subjected to the different breakages during the dissolution process, thus producing a series of polypeptides with different molecular weights. Because small molecular polypeptides are removed during the dialysis with a molecular weight cut-off of 3 kDa, the obvious difference of amino acid compositions appears between rabbit hair and regenerated keratin. Additionally, the low-sulfur, high-sulfur, and high-tyrosine protein fractions of rabbit hair might be ruptured to different extents. This is also the reason for the changes of amino acid compositions.

## 4. Conclusions

Considering that the waste rabbit hair from livestock and textile industries is a valuable keratin source, and that keratins obtained by different dissolution methods have diverse applications, the present study provided an approach to dissolve and extract keratin from rabbit hair using the mixture of choline chloride and oxalic acid at a molar ratio of 1:2 as a DES which is considered to be nontoxic and eco-friendly. The instrumental analyses demonstrated that the disorder of the crystalline structures of protein and the breakage of the disulfide bond of protein chains occurred during the dissolution process. The solubility test revealed that increasing dissolution temperature remarkably enhanced the dissolution of rabbit hair. After dissolution, the pure keratin powder was easily obtained by dialysis, filtration, and freeze-drying. The keratin produced by dissolving in DES at 120 °C displayed flaky powders after freeze-drying and lower thermal stability than the raw rabbit hair, and had a molecular weight ranging from 3.8 to 5.8 kDa. The sum of serine, glutamic acid, cysteine, leucine, and arginine accounted for 59.8 wt % in the total content of analyzed amino acids. The keratin obtained by dissolving rabbit hair in DES could be used as the dyeing and finishing agents for the chemical processing of textiles, and may also find application in areas where a low molecular weight keratin is required.
